# Administrative Data Is Insufficient to Identify Near-Future Critical Illness: A Population-Based Retrospective Cohort Study

**DOI:** 10.3389/fepid.2022.944216

**Published:** 2022-07-25

**Authors:** Allan Garland, Ruth Ann Marrie, Hannah Wunsch, Marina Yogendran, Daniel Chateau

**Affiliations:** ^1^Department of Medicine, University of Manitoba, Winnipeg, MB, Canada; ^2^Department of Anesthesia, University of Toronto, Toronto, ON, Canada; ^3^Manitoba Centre for Health Policy, University of Manitoba, Winnipeg, MB, Canada; ^4^Research School of Population Health, Australian National University, Canberra, ACT, Australia

**Keywords:** critical illness, population health, administrative data, forecasting, cluster analysis, routinely collected health data

## Abstract

**Background:**

Prediction of future critical illness could render it practical to test interventions seeking to avoid or delay the coming event.

**Objective:**

Identify adults having >33% probability of near-future critical illness.

**Research Design:**

Retrospective cohort study, 2013–2015.

**Subjects:**

Community-dwelling residents of Manitoba, Canada, aged 40–89 years.

**Measures:**

The outcome was a near-future critical illness, defined as intensive care unit admission with invasive mechanical ventilation, or non-palliative death occurring 30–180 days after 1 April each year. By dividing the data into training and test cohorts, a Classification and Regression Tree analysis was used to identify subgroups with ≥33% probability of the outcome. We considered 72 predictors including sociodemographics, chronic conditions, frailty, and health care utilization. Sensitivity analysis used logistic regression methods.

**Results:**

Approximately 0.38% of each yearly cohort experienced near-future critical illness. The optimal Tree identified 2,644 mutually exclusive subgroups. Socioeconomic status was the most influential variable, followed by nursing home residency and frailty; age was sixth. In the training data, the model performed well; 41 subgroups containing 493 subjects had ≥33% members who developed the outcome. However, in the test data, those subgroups contained 429 individuals, with 20 (4.7%) experiencing the outcome, which comprised 0.98% of all subjects with the outcome. While logistic regression showed less model overfitting, it likewise failed to achieve the stated objective.

**Conclusions:**

High-fidelity prediction of near-future critical illness among community-dwelling adults was not successful using population-based administrative data. Additional research is needed to ascertain whether the inclusion of additional types of data can achieve this goal.

## Introduction

The care of critically ill people in intensive care units (ICUs) is an important part of healthcare in all industrialized countries. Approximately 0.5–1.3% of all adults are admitted to ICUs every year, which is rising rapidly with age and amounting to 2–5% of people 85 years of age or older (1, 2). In the United States, up to half of all people experience ICU care during their final year of life (3), and many die there (4, 5). In Canada, 11% of hospitalizations include time in an ICU (6) and 19% of people die there (7). Estimates from the United States indicate that ICU care comprises ~4% of total national health expenditures (8), equating to 0.7% of the national gross domestic product (9). Furthermore, ICU utilization is also rising (6, 10). Critical illnesses cause burdens for society by inhibiting the ability of survivors to work and earn (11, 12).

The risk of death from critical illness is high, but mortality is only one of its negative consequences. Many survivors experience ongoing physical, cognitive, and psychological problems (13). It would be a major advance to prevent or delay critical illness among community-dwelling adults. Prospectively identifying adults with a high probability of developing critical illness in the near future is a necessary first step toward designing and testing interventions to achieve this advance. In this work, we specifically sought to identify adults with a >33% probability of near-future critical illness.

For the maximum value, the prediction of critical illness needs to be feasible using readily accessible data that are population-based and available on an ongoing basis. Administrative (health claims) data meet these criteria (14). While previous studies have attempted similar predictions, they have met with limited success (15–20) We hypothesized that applying advanced statistical methods to longitudinal information about medical resource utilization, coupled with information about demographics and serious health conditions, in a pre-COVID-19 era would: (a) identify subgroups with high a probability of near-future critical illness and (b) identify a consequential fraction of all people who develop that outcome.

## Materials and Methods

### Design, Setting, and Data Sources

This retrospective cohort study used administrative health data from the universal, single-payer healthcare system in the Canadian province of Manitoba. Available to all Manitoba residents, this system covers inpatient and outpatient care, practitioner fees, diagnostic testing, long-term care, and homecare. There is limited coverage for outpatient eye examinations, chiropractor, and physical therapy visits. An outpatient prescription drug benefit plan with an income-related deductible is available to low-income registrants. Services not covered include outpatient care by dentists, podiatrists, acupuncturists, psychologists, and dietitians; cosmetic surgery; and ambulance transport, with the exception of air ambulance transport for residents who live north of the 53rd parallel.

The databases used for this study (Supplementary Table 1) are held in the Manitoba Centre for Health Policy Research Data Repository (21). As previously described (22–24), they are linked *via* an anonymized version of the unique Personal Health Identification Number. New data are updated every 6 months, routinely cleaned, and checked. These data have been demonstrated to have high validity and reliability for investigating health and the use of healthcare (22).

The Discharge Abstract Database (DAD) captures detailed data for every hospitalization, including admission and discharge dates, up to 25 diagnoses reported using the International Classification of Disease (ICD)-10th edition Canadian format, and up to 15 procedures in the Canadian Classification of Interventions (CCI) format (25–27). Centrally trained data abstractors working in each acute care hospital collect these data using nationally uniform definitions, format, collection methods, and data entry software (28). DAD data are validated and reported to the Canadian Institute for Health Information by the provincial health authority. The DAD is highly accurate in identifying the delivery and timing of ICU care (29).

This study was approved by the University of Manitoba Health Research Ethics Board and Manitoba's Health Information Privacy Committee.

### Study Population

The source for this study was the Manitoba population (30). We included three fiscal year cohorts (FY2013–2015, each from April 1 to May 30). April 1 was the *start date* of each FY at which inclusion and exclusion criteria were applied. We included individuals aged 40–89 years who were continuously registered with Manitoba Health from 5 years before the start date to the previous 1 year after the start date or the critical illness date, if it occurred.

We excluded individuals who had incident malignancies in the 5-year period preceding the start date; those who were in an acute care facility on the start date; or were enrolled in a palliative care program anytime during the 2 years preceding the start date. The rationale for excluding individuals with incident cancers derives from the fact that since ICU admission and death due to cancers are common (31, 32) and undiagnosed cancers are rare (33, 34), critical illness or death from cancer is unlikely to be avoidable. The 5-year interval is a common benchmark for cancer survival. Generalizing the finding of Lix et al. (35), we identified incident malignancy based on the presence of at least one inpatient or outpatient diagnosis code occurring within 5 years before the start date, and for which no other cancer diagnosis codes were identified during the 5–10 years prior to that code. We used accepted diagnosis codes [Supplementary Table 2; (36)]. We excluded individuals in an acute care facility because our goal was to identify individuals residing in the community who were presumably medically stable when they develop the outcome. Individuals enrolled in palliative care programs were excluded because they have a short life expectancy and would not seek aggressive and curative medical care at the end of life.

### Outcome

Our outcome was a critical illness that occurred in the near future, defined as 30–180 days after the start date. Thirty days was chosen as the lower limit as it would require some time to locate, contact, and engage the individual in an intervention seeking to avoid the adverse outcome. The 180-day upper limit provides sufficient time for outcomes to occur, while expecting that the ability to predict such future events would degrade with the passage of time after the start date.

Following prior work, critical illness was defined as the presence of either of the following events: (i) non-elective hospital admission that included care in a high-intensity ICU with the use of artificial life support, or (ii) non-palliative death, in or out of the hospital (18, 37, 38). For non-elective admissions, we excluded hospitalizations for trauma or injury (Supplementary Table 2), as they are unforeseen and expected to be much more difficult to predict or prevent. The critical illness date was taken as the earlier of the two events within the 30- to 180-day interval. High-intensity ICUs are those capable of providing artificial life support for an unlimited period. During the study period, Manitoba had 10 such adult ICUs serving its population of 1.3 million (30).

Though we sought to include ICU admissions involving the use of any of the three most common types of artificial life support (invasive mechanical ventilation (IMV), intravenous vasoactive drugs, or renal replacement therapies), DAD coding has proved only sufficiently accurate for invasive mechanical ventilation (39). However, 81% of ICU patients in our cohort who received vasoactive drugs or renal replacement therapies were also mechanically ventilated (39).

Invasive mechanical ventilation was identified by CCI procedure codes (Supplementary Table 2). Enrollment in palliative care was defined as any of the following being present in the 2 years before the start date: (i) in palliative care in any Manitoba hospital, identified by the presence of hospital diagnosis coding (Supplementary Table 2); (ii) DAD service codes indicating primary responsibility for hospital care under the palliative care service; (iii) outpatient palliative care identified by palliative care codes in the provincial Home Care database; or (iv) outpatient pharmacy database, indicating medication payment under the provincial palliative care program.

### Analysis

Our primary analysis used Classification and Regression Trees (CART) (40, 41), seeking to identify subgroups of community-dwelling adults who experienced high rates of critical illness 30–180 days after the start date (Appendix A). CART divides a cohort into mutually exclusive subgroups, each defined by a given value/category of each input variable. The result is a ramified tree where each “terminal leaf” includes one homogeneous subgroup. Using CART to identify such individuals amounts to identifying terminal leaves in which a sufficiently high fraction of included persons experience the outcome. We chose 33% as being a sufficiently high fraction as it represents needing to intervene on three people to have a chance of avoiding one outcome.

To create our CART model, we used data from FY2013 and 2014 as the Training data, randomly splitting the data (60:40) into two subcohorts, which were used to train the model. Subsequently, we assessed this model on the FY2015 cohort (Test data). We report the relative influence of each predictor variable in the final tree, calculated such that the top-ranked predictor variable is assigned a value of 1.0 (42). To evaluate predictive ability, we report LIFT (43), defined as the fraction of outcome events in the subgroup(s) divided by the fraction in the originating population. See Appendix A for more information, including the CART settings used.

In a sensitivity analysis, we assessed the performance of logistic regression for predicting the outcome, combining FY2013 and 2014 data for model development, and then applying that model to FY2015 data. All independent variables (next section) were included. Given the low fraction of outcomes, we used Firth's method of bias correction.

For the comparison of parameters between groups, *t*-test, χ^2^ test, or Fisher's exact test were used, as appropriate. All analyses were performed using SAS version 9.2 and SAS Enterprise Miner version 13.5 (SAS Institute Inc., Cary, NC).

### Predictive Factors

We included 72 parameters (included as 93 input variables) encompassing measures of sociodemographics, chronic comorbid conditions, frailty, and prior health care use (Supplementary Table 3). Sociodemographic variables were age, sex, residing in a nursing home, awaiting placement in a nursing home, rurality of living status [assessed by Statistical Area Classification (44)], straight-line distance from residence location to the nearest high-intensity ICU, socioeconomic status [assessed by an area-level measure, the Socioeconomic Factor Index-2 (SEFI-2), where higher values represent lower socioeconomic status (45)], and having ever received public income assistance. Standard coding was used to identify 32 chronic, comorbid conditions (36). Three administrative data measures of frailty were included (46–48).

A motivating concept of this work was that substantial additional power for predicting near-future health events would be derived from longitudinal medical resource use data. For example, over and above the existence of chronic conditions, a pattern of the rapidly rising use of medical resources might indicate a higher risk of near-future critical illness. We, therefore, included longitudinal information about the use of six types of medical care: (i) number of classes of prescription medications dispensed, (ii) hospital days, (iii) days in Alternative Level of Care [awaiting long-term placement] and rehabilitation facilities, (iv) outpatient visits, (v) outpatient laboratory tests performed, and (vi) separate days in which the individual made one or more calls to Manitoba Health Links, a phone-based system available around-the-clock, where registered nurses follow assessment guidelines to triage of health issues (49). We originally planned to identify trajectories of utilization *via* group-based methods (50); however, in our very large cohorts, it proved unable to identify subject subsets which were substantial in absolute numbers, but represented small fractions of the cohort (e.g., <2%, representing 10,000 people within a yearly cohort). Therefore, for each of the six measures, we instead included counts during each of four intervals before the start date: (A) 13–24, (B) 5–12, (C) 4–6, and (D) 0–3 months prior. Although this approach does not explicitly include patterns of use, CART can include counts from different intervals to relate the outcome to temporal patterns of resource use, if present.

Finally, we included the most recent use of intensive care, and three common, invasive diagnostic procedures (cardiac catheterization, upper or lower gastrointestinal endoscopy, bronchoscopy) prior to the start date. These were classified as: 0–1, 2–6, 7–12, 13–24, or >24 months prior to the start date.

## Results

### Study Populations

Approximately 536,000 individuals comprised each of the 3 yearly cohorts (Table 1; Supplementary Table 4). In all three, 0.38% of individuals experienced the outcome. Each CART input variable differed between those who did vs. those who did not experience the outcome, in terms of statistical significance and absolute terms. People with the outcome were 2–9 times more likely to have had ICU care, cardiac catheterization, GI endoscopy, and bronchoscopy within the 1 month before the start date. They were 10–21 times more likely to live in a personal care home or to have an open homecare file. They were over ~2.5-fold more likely to have frailty scores in the highest tercile. In the 3 months prior to the start dates, people with the outcome had, on average, 1.5 more hospital days, 0.8 more outpatient visits, 0.8 more outpatient laboratory tests, and filled prescriptions for 3.4 additional classes of drugs than individuals without the outcome.

**Table 1:** Selected characteristics of the datasets used for analysis (see Supplementary Table 4 for a complete list).

***Variable***	**Training data: 2013–14**†		**Test data:** ***2015***†	
	***(-)Outcome***	***(+)Outcome***	***(-)Outcome***	***(+)Outcome***
*N*	1,060,252	4065 (0.38%)	541,703	2044 (0.38%)
**Outcome breakdown:**
ICU admission with IMV only		649 (16.0)		380 (18.6)
Non-palliative death only		3145 (77.3)		1529 (74.8)
Both		271 (6.7)		135 (6.6)
**Age (yrs)**
40–44	154,117 (14.5)	63 (1.5)	77786 (14.4)	44 (2.2)
45–49	161,566 (15.2)	129 (3.2)	78277 (14.5)	57 (2.8)
50–54	174,946 (16.5)	221 (5.4)	88351 (16.3)	109 (5.3)
55–59	156,373 (14.7)	296 (7.3)	80164 (14.8)	158 (7.7)
60–69	228,638 (21.6)	820 (20.2)	121610 (22.4)	387 (18.9)
70–79	119,940 (11.3)	983 (24.2)	63195 (11.7)	525 (25.7)
80–89	64,672 (6.1)	1553 (38.2)	32320 (6.0)	764 (37.4)
Female sex	544,620 (51.4)	1797 (44.2)	277581 (51.2)	894 (43.7)
Prior income assistance	107133 (10.1)	600 (14.8)	56795 (10.5)	336 (16.4)
**Timing of prior ICU admission**
0–1 months	387 (0.04)	12 (0.3)	189 (0.03)	8 (0.4)
2–6	1764 (0.2)	62 (1.5)	973 (0.2)	24 (1.2)
7–12	2047 (0.2)	46 (1.1)	936 (0.2)	19 (0.9)
13–24	3981 (0.4)	82 (2.0)	1861 (0.3)	31 (1.5)
>24 or none	1,052,073 (99.2)	3863 (95.0)	537744 (99.3)	1962 (96.0)
Open homecare file	11,469 (1.1)	443 (10.9)	5780 (1.1)	227 (11.1)
Lives in long-term care	9233 (0.9)	761 (18.7)	4264 (0.8)	343 (16.8)
Awaiting long-term care placement	583 (0.05)	15 (0.4)	206 (0.04)	8 (0.4)
**Segal frailty score, terciles**
0	353,738 (33.4)	186 (4.6)	181028 (33.4)	110 (5.4)
0.010–0.030	354,929 (33.5)	542 (13.3)	180924 (33.4)	266 (13.0)
0.031–1.00	351,585 (33.2)	3337 (82.1)	179750 (33.2)	1669 (81.7)
**MsIsaac frailty score, terciles**
0–3	341,885 (32.2)	318 (7.8)	188800 (34.9)	202 (9.9)
3.5–5.5	373,570 (35.2)	625 (15.4)	183124 (33.8)	340 (16.6)
6–23	344,797 (32.5)	3122 (76.8)	169778 (31.3)	1503 (73.5)
Dementia	15968 (1.5)	762 (18.8)	8023 (1.5)	348 (17.0)
**Elixhauser comorbidities**
Hypertension without complications	347,428 (32.8)	2419 (59.5)	189505 (35.0)	1311 (64.1)
Depression	232,502 (21.9)	1132 (27.9)	120653 (22.3)	586 (28.7)
Rheumatoid arthritis/CVD	187,681 (17.7)	840 (20.7)	98191 (18.1)	454 (22.2)
Diabetes mellitus without complications	146,011 (13.8)	1286 (31.6)	78753 (14.5)	721 (35.3)
Chronic pulmonary disorders	126,858 (12.0)	1107 (27.2)	68258 (12.6)	550 (26.9)
Hypothyoridism	74,779 (7.1)	420 (10.3)	40957 (7.6)	215 (10.5)
Cardiac arrythmia	41,327 (3.9)	809 (19.9)	21582 (4.0)	408 (20.0)
Deficiency anemia	31,376 (3.0)	351 (8.6)	19401 (3.6)	204 (10.0)
Obesity	29,413 (2.8)	189 (4.7)	15654 (2.9)	105 (5.1)
Other neurologic disorders	27,274 (2.6)	453 (11.1)	14521 (2.7)	226 (11.1)
Congestive heart failure	24,988 (2.4)	917 (22.6)	12649 (2.3)	414 (20.3)
Drug abuse	23,822 (2.3)	135 (3.3)	11165 (2.1)	70 (3.4)
Cancer without metastices	22,118 (2.1)	368 (9.1)	11567 (2.1)	188 (9.2)
Peripheral vascular disease	21,862 (2.1)	433 (10.7)	11383 (2.1)	191 (9.3)
Liver disease	19,310 (1.8)	194 (4.8)	10618 (2.0)	94 (4.6)
Fluid/electolyte disorders	13,979 (1.3)	430 (10.6)	7316 (1.4)	205 (10.0)
Psychosis	13,474 (1.3)	352 (8.7)	7569 (1.4)	163 (8.0)
Coagulopathy	12,663 (1.2)	193 (4.8)	6290 (1.2)	91 (4.5)
Renal disease	12,558 (1.2)	422 (10.4)	6600 (1.2)	204 (10.0)
Diabetes mellitus with complications	12,353 (1.2)	485 (11.9)	6193 (1.1)	238 (11.6)
Valvular heart disease	10,449 (1.0)	197 (4.9)	5301 (1.0)	100 (4.9)
Peptic ulcer disease without bleeding	7570 (0.7) ^a^	63 (1.6)	3744 (0.7)	47 (2.3)
Alcohol abuse	7454 (0.7)	138 (3.4)	3521 (0.6)	67 (3.3)
Paraplegia/hemiplegia	4097 (0.4)	81 (2.0)	1955 (0.4)	42 (2.1)
Pulmonary circulatory disorders	4039 (0.4)	99 (2.4)	2047 (0.4)	45 (2.2)
Lymphoma	2145 (0.2)	57 (1.4)	1156 (0.2)	30 (1.5)
Hypertension with complications	1759 (0.2)	44 (1.1)	1159 (0.2)	21 (1.0)
Metastatic cancer	1235 (0.1)	105 (2.6)	721 (0.1)	47 (2.3)
Weight loss	1149 (0.1)	52 (1.3)	596 (0.1)	14 (0.7)
HIV/AIDS	809 (0.08) ^b^	8 (0.2)	642 (0.1) ^c^	6 (0.3)
Blood loss anemia	283 (0.03)	15 (0.4)	121 (0.0)	7 (0.3)
**Hospital-days** ^‡^
A, mean ± SD	0.41 ± 3.67	4.08 ± 12.80	0.35 ± 3.25	3.48 ± 10.85
B, mean ± SD	0.22 ± 2.44	2.22 ± 8.69	0.20 ± 2.32	2.20 ± 9.06
C, mean ± SD	0.11 ± 1.61	1.37 ± 6.41	0.11 ± 1.58	1.52 ± 6.53
D, mean ± SD	0.097 ± 1.38	1.47 ± 5.87	0.10 ± 1.41	1.77 ± 6.58
**Outpatient clinic visits** ^‡^
A, mean ± SD	5.2 ± 5.5	7.7 ± 7.3	5.4 ± 5.5	8.2 ± 7.3
B, mean ± SD	2.8 ± 3.1	4.3 ± 4.4	2.8 ± 3.1	4.4 ± 4.2
C, mean ± SD	1.3 ± 1.7	2.1 ± 2.4	1.4 ± 1.7	2.1 ± 2.4
D, mean ± SD	1.3 ± 1.7	2.2 ± 2.4	1.3 ± 1.7	2.1 ± 2.5
**Outpatient laboratory test counts** ^‡^
A, mean ± SD	4.7 ± 7.5	7.1 ± 11.7	5.0 ± 7.9	7.6 ± 11.7
B, mean ± SD	2.5 ± 4.8	4.0 ± 7.2	2.7 ± 5.1	4.2 ± 7.4
C, mean ± SD	1.2 ± 3.1	1.9 ± 4.2	1.3 ± 3.3	1.9 ± 4.1
D, mean ± SD	1.2 ± 3.1	2.1 ± 4.3	1.4 ± 3.3	2.1 ± 4.5
**ATC4 prescription counts** ^‡^
A, mean ± SD	3.6 ± 3.8	7.6 ± 5.4	3.6 ± 3.8	7.5 ± 5.3
B, mean ± SD	2.8 ± 3.1	6.3 ± 4.6	2.8 ± 3.2	6.3 ± 4.5
C, mean ± SD	2.3 ± 2.8	5.5 ± 4.2	2.3 ± 2.8	5.5 ± 4.1
D, mean ± SD	2.3 ± 2.8	5.6 ± 4.3	2.3 ± 2.9	5.7 ± 4.3

*Values are # (%) unless indicated otherwise. All p-values< 0.0001 for differences between subjects with vs. without the outcome, except as indicated. †Fiscal years (April 1-May 30); Timing backward from the Start date: (A) 13–24 months prior, (B) 5–12 months prior, (C) 4–6 months prior, (D) 0–3 months prior; ICU, intensive care unit; IMV, invasive mechanical ventilation; ATC4, the fourth level of the Anatomic Therapeutic Chemical Classification system; CVD, collagen-vascular diseases*;

*^a^p = 0.014*;

*^b^p = 0.006; ^c^p = 0.02*.

### CART Analysis

The final optimal tree had 30 levels of branching and 2,644 terminal leaves (Supplementary Table 5). The initial branch point was by residence in a nursing home. All subjects residing in nursing homes as of the start date were included in a single terminal leaf; the larger (60%) subcohort of the Training data contained 5,954 subjects, of whom 470 (7.9%) experienced the outcome. Appendix B contains an example of how CART can combine input variables in complex combinations.

The input variable with the highest predictive value was socioeconomic status, followed by living in a nursing home (Table 2; Supplementary Table 6). The Segal and McIsaac frailty measures occupied the third and fifth slots, and age was sixth. Utilization of outpatient care and drug prescriptions were the highest-ranked parameters of medical resource use; though generally counts further back in time from the start date were more influential than were those that were closer to the start date. The first appearance of a count of hospital days is in the 14th slot, with relative importance less than half that of socioeconomic status. Among the 32 specific chronic diagnoses, all had importance values <0.29 on this relative scale ranging from 0 to 1.

**Table 2:** Relative predictive value of top 25 variables in the optimal Classification and Regression Tree solution (see Supplementary Table 6 for a complete list).

	**Input variable**	**Relative importance**
1	Socioeconomic status (SEFI-2)	1.000 (reference)
2	Lives in long-term care	0.883
3	Segal frailty score	0.879
4	Distance from home to closest high-intensity	0.860
5	McIsaac frailty score	0.810
6	Age	0.756
7	Outpatient clinic visits: 7–12 months prior to start date	0.587
8	Outpatient clinic visits: 13–24 months prior to start date	0.580
9	Outpatient laboratory test counts: 13–24 months prior to start date	0.577
10	ATC4 prescription counts: 7–12 months prior to start date	0.558
11	ATC4 prescription counts: 4–6 months prior to start date	0.549
12	ATC4 prescription counts: 13–24 months prior to start date	0.535
13	ATC4 prescription counts: 0–3 months prior to start date	0.504
14	Hospital-days: 13–24 months prior to start date	0.458
15	Outpatient laboratory test counts: 7–12 months prior to start date	0.449
16	Outpatient clinic visits: 4–6 months prior to start date	0.444
17	Outpatient clinic visits: 0–3 months prior to start date	0.421
18	Outpatient laboratory test counts: 4–6 months prior to start date	0.416
19	Hospital-days: 0–3 months prior to start date	0.401
20	Hospital-days: 7–12 months prior to start date	0.383
21	Hospital-days: 4–6 months prior to start date	0.301
22	Metastatic cancer	0.285
23	Outpatient laboratory test counts: 4–6 months prior to start date	0.274
24	Statistical area classification	0.200
25	Open homecare file	0.186

*ATC4, fourth level of the Anatomic Therapeutic Chemical Classification system; SEFI, socioeconomic factor index*.

In the Training data, the optimal tree performed well in identifying individuals with the outcome (Table 3); 493 subjects contained in 41 terminal leaves each had ≥33% of its members with the outcome. However, most of this performance in predicting near-future critical illness represented overfitting of the model to the Training data, as this performance was not reproduced when applying the same terminal leaf definitions to the Test data (Table 3). In the Test data, these 41 leaves contained 429 individuals, but only 20 (4.7%) of them had the outcome, representing 0.98% of all those with the outcome. Expanding the range of terminal leaves in the Training data to those with ≥ 20% or ≥10% outcomes likewise performed well in the Training data, but this was not reproduced in the Test data (Table 3).

**Table 3:** Performance of the optimal Classification and Regression Tree in identifying individuals with the outcome.

	**Outcome percentages in training dataset**					
	**≥33%** **(41 leaves)**		**≥20%** **(279 leaves)**		**≥10%** **(863 leaves)**	
	**Training data** ^**‡**^	**Test data**	**Training data** ^**‡**^	**Test data**	**Training data** ^**‡**^	**Test data**
# Subjects indicated by the model	493	429	3,242	2,871	9,849	9,111
# Of those with the outcome	191	20	832	100	1,632	171
As % of subjects indicated by the model	38.7	4.7	25.7	3.5	16.6	1.9
As % of all subjects with the outcome	7.8	0.98	34.2	4.9	66.8	8.4
Lift^†^	102.0	12.3	67.5	9.2	43.6	4.9

*Quotient of outcome events in the designated leaves, and in the originating population*;

*^‡^Values are for the larger (60%) random subcohort of the fiscal year 2013 and 2014 Training dataset*.

### Sensitivity Analysis

In the sensitivity analysis, unlike CART, logistic regression modeling performed similarly in the Training and Test data (Table 4). Although the Test data logistic modeling correctly identified a larger percentage of those flagged as having ≥33% probability of the outcome (20.5 vs. 4.7% from CART), it identified a similarly low percentage of all those with the outcome (1.1 vs. 0.98% for CART).

**Table 4:** Performance of logistic regression model in identifying individuals with the outcome.

	**Predicted outcome percentage cutoff**					
	**≥33%**		**≥20%**		**≥10%**	
	**Training data**	**Test data**	**Training data**	**Test data**	**Training data**	**Test data**
# Subjects indicated by the model	298	112	1,030	464	3,830	1,760
# Of those with the outcome	56	23	139	71	462	202
As % of subjects indicated by the model	18.8	20.5	13.5	15.3	12.1	11.5
As % of all subjects with the outcome	1.4	1.1	3.4	3.5	11.4	9.9

## Discussion

High-fidelity prediction of a substantial fraction of persons experiencing near-future critical illness was not possible using administrative healthcare data alone. Specifically, we did not succeed in prospectively identifying a substantial number of individuals belonging to subgroups of community-dwelling Manitobans with a ≥33% probability of developing critical illness in the following 6 months. We chose the 33% threshold to make it practical to design and test specially designed interventions seeking to avoid or delay the coming health event, assuming that these interventions would be resource-intensive. However, for individuals in those subgroups in our future (Test) data, that parameter was 4.7% and not 33%, and comprised ~1% of all those with the outcome. While applying logistic regression to these administrative data showed less overfitting compared to CART, it likewise failed to achieve the stated objective.

Two prior efforts sought to predict future critical illness among unselected, community-dwelling persons (16, 18). Neither were population-based; both used logistic regression with fewer input variables than our study. Among 4.7 million health plan enrollees in a validation cohort (16), 0.75% experienced ICU admission within the following 1 year. Among the 1% of subjects with the highest predicted risk, 35% experienced the outcome, though this represented only 0.49% of all those with the outcome. In comparison, 0.38% of our validation cohort experienced our outcome within 180 days, and among those with predicted risk exceeding 33%, 4.7% experienced the outcome, representing 0.98% of all those with the outcome. In what was evidently a very different substrate, among 9,742 people 65 years and older attending Mayo Clinic outpatient clinics, 8.8% in the cohort experienced critical illness within the following 2 years, and among the 11% with the highest risk score, 26% experienced the outcome, which was 33% of those with the outcome (18). Other studies have used regression methods but for different goals, including attempts to predict future critical illness among patients brought to hospital *via* ambulance, hospitalization and/or death among community-dwelling persons, and future need for mechanical ventilation among community-dwelling persons (15, 17, 37, 51). It is important to note that although efforts to identify people at high risk of outcomes, such as future critical illness or death have reported good results using the c-statistic as the metric (52), the c-statistic failure is inappropriate for a purpose such as ours because it fails to account for the underlying prevalence of the disorder of interest (53).

Potential methodologic limitations deserve discussion. We included numerous input variables representing a wide variety of concepts related to health and health care, including the novel aspect of incorporating prior medical resources in a way that allowed for accounting for trajectories of use. We did not include other administrative data such as immunizations, immigrant status, education, Emergency Department visits, or results of historical laboratory tests. While such additional information could plausibly add predictive power, it added only a small increment in an analysis of 1-year mortality among hospitalized patients (20). Second, we used CART analysis, a flexible and powerful statistical method that allows for arbitrarily complicated interactions among the input variables. Sensitivity analysis using logistic regression modeling likewise failed to achieve our goal. While it is possible that another machine-learning method might perform better, direct comparisons across a variety of clinical areas have not found any method to be consistently superior (54–58) Furthermore, a recent systematic review directly comparing machine learning methods to logistic regression reported no significant differences in predictive performance among studies with methodology at low risk of bias (59). Third, our choice of 30–180 days forward from the start date as constituting the “near future” was chosen *a priori*, but could be questioned. Fourth, we chose a composite outcome that included non-palliative, non-ICU death. Recalling that we sought to identify critical illness that could be anticipated and possibly delayed or avoided, this composite derives from the following: (i) the idea that any death is associated with critical illness, even if that illness was very brief, by recognizing that if such a person had been close to death at the time of discovery, rather than being dead, they might have survived long enough to be admitted to an ICU; and (ii) including them helps address the facts that economically disadvantaged persons and those in remote communities have less access to timely care, causing higher rates of prehospital death. This concept has been previously used in assessing disparities in access to ICU care and found to demonstrate reassuring face validity (38). Previous studies have also included death as part of critical illness in prediction efforts (18, 37), though they did not distinguish between palliative and non-palliative deaths. A limitation of this concept is the inability to identify individuals who do not desire or receive ICU admission when they become critically ill but lack formal identification of palliative care. This describes many residents of nursing homes, who have standing Do Not Resuscitate orders but are not enrolled in formal palliative care programs. It would be best to identify such individuals and exclude them from our cohort; however, our data do not contain the information needed to do so. Their inclusion likely introduced misclassification in our outcome, potentially reducing the performance of our predictive model. Fifth, slight differences in predictive performance may have occurred by limiting critical illness onset from April 1 to September 30. Sixth, we excluded patients hospitalized for trauma; however, as we had no direct information about prehospital trauma deaths, we were unable to exclude them from our cohort. Combining Canadian age- and cause-specific death data (32, 60) with the knowledge that 51% of trauma deaths in our included age group occur prehospital (61), we estimate 119 such deaths yearly, indicating a 5.8% overestimation of the number of yearly outcomes experienced in our cohorts. Finally, an explanation for reporting on older data is provided in Appendix C.

The predictive importance of frailty was notable. Frailty may be defined as a “syndrome of age-related physiological decline, characterized by marked vulnerability to adverse health outcomes” (62); it is associated with mortality and morbidity, and with a reduced ability to benefit from aggressive medical interventions. The two predominant formal ways of measuring frailty relate to functioning (63, 64). As administrative data does not contain such information, claims-based frailty measures utilize surrogate parameters and/or lists of comorbid conditions (46–48). In light of this limitation, we chose to include three different administrative data measures of frailty. In our analysis, the frailty administrative data definitions of Segal et al. (47) and McIsaac et al. (48) were among the five most influential variables, indicating some non-overlap between what they are capturing. That they had relative importance almost 3-fold higher than even the most influential specific chronic condition (metastatic cancer) suggests, as have some prior findings (65), that much of the influence of chronic conditions on future outcome may be mediated by the frailty they cause, rather than the condition *per se*.

Although longitudinal measures of medical resource use were influential input variables for predicting the outcome, it was generally not their most recent values that were most important. This may indicate that our outcome relates more to longer-term processes than recent/sudden changes, and it may, in part, explain the poor performance of prior attempts to predict future clinical outcomes based primarily on recent data (18, 37, 66).

We are led to a potentially important hypothesis from the failure of studies including ours and the others mentioned above (16, 18, 20), to accurately predict future medical needs or outcomes. That hypothesis is that high-fidelity prediction, if possible at all, will require the inclusion of input parameters that tap into different types of information than do administrative and clinical data; these may include innate biologies such as genetics and epigenetics, health behaviors, environmental exposures, and other socioeconomic factors. We conclude that to achieve high fidelity prediction of future critical illness, it is necessary to go back to the basics and develop a stronger conceptual framework to help identify the full range of variables that might be influential and to determine how they may be routinely captured at the population level.

## Data Availability Statement

The datasets presented in this article were derived from administrative health data as a secondary use. The data custodian is Manitoba Health and Seniors Care, and were provided under specific data sharing agreements only approved for use at the Manitoba Centre for Health Policy. Where necessary, source data specific to this article or project may be reviewed at the Manitoba Centre for Health Policy with the consent of the original data providers along with the required privacy and ethical review bodies. Requests to access these datasets should be directed to Charles Burchill, charles_burchill@cpe.umanitoba.ca.

## Ethics Statement

This study was approved by the University of Manitoba Health Research Ethics Board and Manitoba's Health Information Privacy Committee. Written informed consent from the patients was not required as it entirely used existing, de-identified data.

## Author Contributions

Conceptualization and funding acquisition: AG. Data curation: AG, MY, and DC. Methodology, writing, review, editing, final approval, and formal analysis: all authors.

## Funding

This work was supported by Manitoba Health and Seniors Care, which had no role in study design, analysis, data interpretation, writing, or the decision to submit. Manitoba Health is the provincial health authority and is the custodian of the data used for this study.

## Conflict of Interest

The authors declare that the research was conducted in the absence of any commercial or financial relationships that could be construed as a potential conflict of interest.

## Publisher's Note

All claims expressed in this article are solely those of the authors and do not necessarily represent those of their affiliated organizations, or those of the publisher, the editors and the reviewers. Any product that may be evaluated in this article, or claim that may be made by its manufacturer, is not guaranteed or endorsed by the publisher.
