# Supplementary material for: Administrative Data Is Insufficient to Identify Near-Future Critical Illness: A Population-Based Retrospective Cohort Study
Source: Front Epidemiol. 2022 Jul 25;2:944216. doi: 10.3389/fepid.2022.944216 (PMC10910992; doi:10.3389/fepid.2022.944216)
Supplement: Supplementary file 1 [file Table_1.pdf]

SUPPLEMENTARY MATERIAL

Administrative Data is Insufficient to Identify Near-Future Critical Illness: A Population-based Retrospective Cohort Study

Garland, Marrie, Wunsch, Yogendran, Chateau

TABLE OF CONTENTS	Pages
Appendix A: Classification and regression tree methodology	1
Appendix B: Example of the ability of CART to combine input variables	2
Appendix C: Explanation for age of data reported	2
Supplementary Table 1: Databases and data elements used	3
Supplementary Table 2: Diagnostic and Procedure Codes Used	3
Supplementary Table 3. Input variables for Classification and Regression Tree (CART) analysis	4-5
Supplementary Table 4. Characteristics of the datasets used for analysis	6-10
Supplementary Table 5. Optimal CART tree branching	11
Supplementary Table 6. Relative predictive value of all input variables in the optimal CART tree solution	12-13
Supplemental References	14

APPENDIX A CLASSIFICATION AND REGRESSION TREE (CART) METHODOLOGY

CART[1,2] is an extremely flexible type of decision tree algorithm that can take account of a very large number of independent variables, automatically allowing for arbitrarily complicated interactions among the them. CART uses recursive partitioning to divide all members of a cohort into mutually exclusive subgroups, each defined by a given value/range/category of the independent variables. The result is a ramified tree where each "terminal leaf" is one such subgroup.

An innate feature of CART is its ability to completely separate subgroups. Identifying the predictors of one subgroup does not hinder its ability to identify those for other subgroups, whether overlapping or completely separate. This is not the case for multivariable regression, where in the absence of explicitly including numerous interaction terms which quickly become difficult to interpret, inclusion of a subgroup (e.g. nursing home residents) influences the coefficients of all other predictors.

We chose to use CART over other classification methods expecting that identifying a substantial number of individuals who will develop critical illness in the near future would require finding a large number of diverse subgroups (represented by terminal leaves) in which >33% experienced the outcome. Such subgroups would require applying an eventual intervention to no more than three people to have a chance to avert or delay one episode of critical illness.

For a binary outcome such as ours, CART seeks a solution maximizing the "purity" of leaves, in the sense that leaves have as great a fraction of zeros or ones as possible;[3] a variety of different measures can be used as the measure of purity. CART grows trees in single steps, working on an existing node and seeking a single variable (and it's splitting value) that improves the purity of the daughter nodes over the parent node that was split. Input variables may be split once, multiple times, or not at all. Conditions that terminate splitting are down a tree branch are: perfect purity is achieved; the chosen maximum number of branches is reached; the chosen minimum leaf size is reached for each leaf; or the chosen threshold p-value for continued splitting is exceeded.

CART uses two subcohorts to Train the model. We created these two portions of the Training dataset by a 60:40 random subdivision of the FY2013 and 2014 data. The first of these subcohorts is used to generate the full, maximal tree, and then the second subcohort is uses to identify the optimal subtree. Identifying the single best subtree begins with the original (maximal) tree of M leaves. By removing individual leaves from the end back towards the origin ("pruning"), it creates all subtrees of leaf number M-1, M-2, M-3, etc. For every such subtree it calculates the *worth* of the tree using one of a number of possible parameters. Using that measure of worth, it chooses the subtree of the highest worth containing M, M-1, M-2, M-3, etc. leaves. Among this family of optimal subtrees of different size, it chooses as the final tree the one that has a higher worth than any smaller tree, but equal or higher worth than any larger tree.

We used *Lift* as the measure of tree worth.[3] Lift measures performance of a tree at predicting events in a chosen subset of leaves, compared to the rate of events in the entire sample. For example, if the overall population rate of events is 1%, but in a given subset of leaves the rate is 20%, then the Lift for this subset is 20.

The CART settings we used in SAS Enterprise Miner were: (a) minimum leaf size = 10, (b) a maximum of 30 levels of branching, (c) 2-way and 3-way branching allowed, (d) splitting criterion was GINI,[3] along with a threshold p-value of 0.20 using both Bonferroni and depth adjustments, (d) tree worth assessed via the Lift measure.[3]

APPENDIX B: EXAMPLE OF THE ABILITY OF CART TO COMBINE INPUT VARIABLES

An example of how CART can combine input variables in complex combinations is the following terminal leaf. This subgroup comprised subjects who were

- female
- living <105 km from the closest high-intensity ICU
- SEFI ≥ -0.65
- 2 clinic visits in the 3 months prior to the Start Date
- hospital-days were:
  - <25 in the most recent 3 months
  - <25 in the prior 7-12 months
  - ≥10 from 13-24 months prior
- ALC + rehabilitation-days were:
  - <52 from 13-24 months prior
  - <42 from 7-12 months prior
- outpatient laboratory tests were:
  - <14 outpatient laboratory tests performed in the most recent 3 months
  - <21 in the prior 7-12 months
- prescriptions: 12-13 different chemical classes filled in the 4-6 months prior.

APPENDIX C: EXPLANATION FOR AGE OF DATA REPORTED

This study was planned and funded in 2019. That plan included creating the CART model using two years of Training data (FY2013/14 and 2014/15), and evaluating its ability for high-fidelity prediction of critical illness in the Test data of subsequent years (FY2015/16, 2016/17 and 2017/18, the latest data then available). Anticipating that our hypothesis was correct, the plan included assessing how the predictive ability decays over time by serially assessing prediction for FY2015/16, then FY2016/17 and finally FY2017/18. However, contrary to our hypothesis, even prediction in the first Test year data (FY2015/16) was unsuccessful. Therefore, assessment of prediction for the two following fiscal years was judged to be futile.

While in principal it would have been possible to redo the analysis using FY2015/16 and FY2016/17 as the Training data and the final year of data (FY2017/18) as the Test year, we chose not to do this for three reasons. First, we considered it very unlikely that doing so would alter the conclusions of the presented manuscript. The others were a confluence of: (ii) analysis delays of approximately nine months over 2020 and 2021 due to the COVID-19 pandemic in Canada, and (iii) the desire of the provincial health department that the report they funded in 2019 would be completed and presented without further delay.

Supplementary Table 1. Databases and data elements used.

Database	***Data elements used***
Manitoba Health Insurance Registry	Presence in Manitoba, age, sex, postal code, marital status, death
Discharge Abstract Database (DAD)	Hospital abstracts: admission/discharge timing, ICU use, diagnoses, procedures, length of stay, alternative level of care length of stay, paneling for nursing home
National Rehabilitation Reporting System	Admission and discharge timing of inpatient rehabilitation services
Medical Services	Outpatient claims: timing, diagnoses, procedures
Diagnostic Services Manitoba	Outpatient laboratory testing
Long-Term Care	Nursing home residency
Home Care	Dates of service, use of home oxygen
Drug Program Information Network (DPIN)	Outpatient prescription drugs dispensed
Social Allowances Management Information Network (SAMIN)	Income assistance history
Health Links	Telephone calls made to this outpatient triage service
Public use Census files	Area-level socioeconomic variables

Supplementary Table 2. Diagnostic and Procedure Codes Used

Entity	***Diagnosis or Procedure codes***
Mechanical ventilation (CCI)	1.GZ.31.CA-ND, 1.GZ.31.CR-ND
Palliative care (ICD-10-CA)	Z51.5
Malignancy	
ICD-9-CA	140.x-172.x, 174.x-195.8, 200.x-208.x, 238.6
ICD-10-CA	C00.x-C26.x, C30.x-C34.x, C37.x-C41.x, C43.x, C45.x-C58.x, C60.x-C76.x, C81.x-C85.x, C88.x, C90.x-C97.x
Trauma, injury (ICD-10-CA)	S00 - T35, T66 - T79, V, W, or X: with diagnosis type indicating present at hospital admission

CCI, Canadian Classification of Interventions

Supplementary Table 3. Input variables for Classification and Regression Tree (CART) analysis

Input Parameter<br>[# concepts/# variables in analysis]	*Form in modeling*	*Notes*
Age [1/1]	continuous	
Sex [1/1]	binary	
SEFI-2 score[4] [1/1]	continuous	-area-level measure of socioeconomic status
Ever received provincial income assistance [1/1]	binary	
Statistical Area Classification type[5] [1/6]	indicator variables for: census metropolitan area, census area, strong metropolitan influenced zone,<br>moderate metropolitan influenced zone, weak metropolitan influenced zone, and no metropolitan influenced zone	-assesses rurality; relates to population density
Distance from residence to closest high-intensity ICU [1/1]	continuous	
Resides in a nursing home [1/1]	binary	
Awaiting opening in a nursing home [1/1]	binary	
Open Homecare file [1/1]	binary	
Chronic medical conditions [32/32]	-binary; 31 per Elixhauser *et al.*[6], and dementia; codes per Quan *et al.*[7]<br>-derived from inpatient and outpatient claims data, up to 24 months prior to the Start Date	
Timing of most recent prior ICU admission [1/5]	-indicator variables for 0-1 month, 2-6 months, 7-12 months, 13-24 months, or >24 months	
Timing of the most recent cardiac catheterization [1/5]	-indicator variables for 0-1 month, 2-6 months, 7-12 months, 13-24 months, or >24 months<br>-from Manitoba physician tariff codes 2302, 2304, 2306, 2307, 2308, 2325, 2327, 2234, 2305, 6263, 6264, 6267,<br>6268, 6270, 6278, 6279, 6280	
Timing of the most recent upper or lower gastrointestinal endoscopy [1/5]	-indicator variables for 0-1 month, 2-6 months, 7-12 months, 13-24 months, or >24 months<br>-from Manitoba physician tariff codes 3055, 3063, 3057, 3065, 3121, 3122, 3123, 3190, 3192, 3095, 3092, 3505, 3506,<br>3498, 3185, 3186, 3187, 3189, 3188, 3196, 3311, 3313, 3315, 3317, 3319, 3320, 3323, 3324, 3312, 3000, 3002, 3004,<br>3010, 3012, 3013	
Timing of the most recent bronchoscopy [1/5]	-indicator variables for 0-1 month, 2-6 months, 7-12 months, 13-24 months, or >24 months<br>-from Manitoba physician tariff codes 2113, 2121, 2126, 2112, 2120, 2115, 2117, 2118	
Segal *et al*. frailty score[8] [1/1]	continuous (ranges 0-1)	-for use with administrative data
McIsaac *et. al.* frailty score[9] [1/1]	continuous (ranges 0-30)	-for use with administrative data<br>-with modifications[a]

ADG frailty indicator[10] [1/1]	binary	-from proprietary, Johns Hopkins ADG system
# of hospital-days during 2 years prior to Start Date [4/4]	divided into 4 variables (A) 13-24 months prior, (B) 5-12 months prior, (C) 4-6 months prior, (D) 0-3 months prior	with exclusions[b]
# of other days in hospital or rehabilitation during 2 years prior to Start Date [4/4]	divided into 4 variables: (A) 13-24 months prior, (B) 5-12 months prior, (C) 4-6 months prior, (D) 0-3 months prior	"ALC/rehab" days[c]
# of outpatient visits during 2 years prior to Start Date [4/4]	divided into 4 variables: (A) 13-24 months prior, (B) 5-12 months prior, (C) 4-6 months prior, (D) 0-3 months prior	-visits with physician, nurse practitioner, primary care nurse<br>-with exclusions[d]
# of outpatient laboratory tests during 2 years prior to Start Date [4/4]	divided into 4 variables: (A) 13-24 months prior, (B) 5-12 months prior, (C) 4-6 months prior, (D) 0-3 months prior	-see note[e]
# of classes of prescription medications dispensed during 2 years prior to Start Date [4/4]	divided into 4 variables: (A) 13-24 months prior, (B) 5-12 months prior, (C) 4-6 months prior, (D) 0-3 months prior	-classified by ATC4 level [11]<br>-see note[f]
# of days with calls to *Manitoba HealthLinks* during 2 years prior to Start Date [4/4]	divided into 4 variables: (A) 13-24 months prior, (B) 5-12 months prior, (C) 4-6 months prior, (D) 0-3 months prior	-call-in, 24-hour, nurse-staffed, provincial triage system[12]

[a] Excluded the perioperative risk portion (HOMR); administrative data definitions of 5 chronic conditions from the Ontario Institute for Clinical Evaluative Sciences were replaced by parallel definitions from the Manitoba Centre for Health Policy

[b] Excludes: scheduled hospital admissions, admissions for routine obstetrical delivery, hospital days awaiting long-term care placement

[c] Includes: days in acute care hospital awaiting long-term care placement, and days in a rehabilitation facility

[d] Excludes: outpatient surgeries; routine obstetrical visits; visits to pediatricians, radiologists, pathologists, anaesthesiologists, emergency medicine physicians, optometrists, chiropracters, midwives; visits on the same day in which the individual was hospitalized. Multiple tariffs submitted on the same date by the same practitioner were counted as a single visit.

[e] Groups of tests usually done altogether were counted as a single test (blood counts, lipids, basic serum electrolyte panel, extended serum electrolytes, urinary electrolytes, serum B12 and folate, liver panel, thyroid panel, coagulation panel, urinalysis, intradermal allergy panel, allergy patch test panel, portions of individual cultures with or without sensitivity testing). Excludes tests performed the same day in which the individual was hospitalized, and testing done during Emergency Department visits.

[f] Two agents of the same chemical class counted as one prescription, regardless of the number of refills in that period, or the number of pills dispensed. Multiple prescriptions for the same ATC4 level of agent filled in the same time interval counted as one prescription

Supplementary Table 4. Characteristics of the datasets used for analysis. Values are # (%) unless indicated otherwise.

Variable	**Training Data: 2013-14†**			**Test Data: *2015*†**		
	(-)Outcome	***p-value***	***(+)Outcome***	***(-)Outcome***	***p-value***	***(+)Outcome***
N	1,060,252		4065 (0.38%)	541,703		2044 (0.38%)
Outcome breakdown:						
ICU admission with IMV only			649 (16.0)			380 (18.6)
Non-palliative death only			3145 (77.3)			1529 (74.8)
Both			271 (6.7)			135 (6.6)
Age (yrs)						
40-44	154,117 (14.5)		63 (1.5)	77786 (14.4)		44 (2.2)
45-49	161,566 (15.2)		129 (3.2)	78277 (14.5)		57 (2.8)
50-54	174,946 (16.5)	<.0001	221 (5.4)	88351 (16.3)	<.0001	109 (5.3)
55-59	156,373 (14.7)		296 (7.3)	80164 (14.8)		158 (7.7)
60-69	228,638 (21.6)		820 (20.2)	121610 (22.4)		387 (18.9)
70-79	119,940 (11.3)		983 (24.2)	63195 (11.7)		525 (25.7)
80-89	64,672 (6.1)		1553 (38.2)	32320 (6.0)		764 (37.4)
Female sex	544,620 (51.4)	<.0001	1797 (44.2)	277581 (51.2)	<.0001	894 (43.7)
Prior income assistance	107133 (10.1)	<.0001	600 (14.8)	56795 (10.5)	<.0001	336 (16.4)
Timing of prior ICU admission						
0-1 months	387 (0.04)		12 (0.3)	189 (0.03)		8 (0.4)
2-6	1764 (0.2)	<.0001	62 (1.5)	973 (0.2)	<.0001	24 (1.2)
7-12	2047 (0.2)		46 (1.1)	936 (0.2)		19 (0.9)
13-24	3981 (0.4)		82 (2.0)	1861 (0.3)		31 (1.5)
>24 or none	1,052,073 (99.2)		3863 (95.0)	537744 (99.3)		1962 (96.0)
Timing of prior cardiac catheterization						
0-1 months	591 (0.06)		14 (0.3)	307 (0.06)		8 (0.4)
2-6	2912 (0.3)	<.0001	53 (1.3)	1471 (0.3)	<.0001	21 (1.0)
7-12	3363 (0.3)		42 (1.0)	1682 (0.3)		25 (1.2)
13-24	6430 (0.6)		63 (1.6)	3053 (0.6)		29 (1.4)
>24 or none	1,046,956 (98.8)		3893 (95.8)	535190 (98.8)		1961 (95.9)
Timing of prior GI endoscopy						
0-1 months	5296 (0.5)	<.0001	50 (1.2)	3108 (0.6)	<.0001	25 (1.2)
2-6	25280 (2.4)		177 (4.4)	12932 (2.4)		86 (4.2)
7-12	27951 (2.6)		133 (3.3)	13792 (2.5)		71 (3.5)

13-24	49853 (4.7)		232 (5.7)	25307 (4.7)		126 (6.2)
>24 or none	951,872 (89.8)		3473 (85.4)	486564 (89.8)		1736 (84.9)
Timing of prior bronchoscopy						
0-1 months	64 (0.01)	<.0001	s	52 (0.01)	<.0001	0 (0.0)
2-6	392 (0.04)		17 (0.4)	178 (0.03)		7 (0.3)
7-12	438 (0.04)		s	194 (0.04)		6 (0.3)
13-24	811 (0.08)		16 (0.4)	374 (0.07)		6 (0.3)
>24 or none	1,058,547 (99.8)		4021 (98.9)	540905 (99.9)		2025 (99.1)
SEFI-2 (octiles)						
lowest (-5.2 to -1.1)	132,693 (12.5)	<.0001	323 (7.9)	67814 (12.5)	<.0001	140 (6.8)
2nd (-1.0 to -0.68)	132,829 (12.5)		338 (8.3)	67796 (12.5)		201 (9.8)
3rd (-0.67 to -0.43)	132,447 (12.5)		443 (10.9)	67740 (12.5)		207 (10.1)
4th (-0.42 to -0.22)	132,853 (12.5)		602 (14.8)	67409 (12.4)		296 (14.5)
5th (-0.21 to 0.02)	132,394 (12.5)		474 (11.7)	67859 (12.5)		255 (12.5)
6th (0.03 to 0.24)	132,230 (12.5)		588 (14.5)	67811 (12.5)		291 (14.2)
7th (0.25 to 0.67)	132,478 (12.5)		596 (14.7)	67649 (12.5)		300 (14.7)
highest (0.68 to 3.88)	132,328 (12.5)		701 (17.2)	67625 (12.5)		354 (17.3)
Open homecare file	11,469 (1.1)	<.0001	443 (10.9)	5780 (1.1)	<.0001	227 (11.1)
Lives in long-term care	9233 (0.9)	<.0001	761 (18.7)	4264 (0.8)	<.0001	343 (16.8)
Awaiting long-term care placement	583 (0.05)	<.0001	15 (0.4)	206 (0.04)	<.0001	8 (0.4)
Segal frailty score, terciles						
0	353,738 (33.4)	<.0001	186 (4.6)	181028 (33.4)	<.0001	110 (5.4)
0.010-0.030	354,929 (33.5)		542 (13.3)	180924 (33.4)		266 (13.0)
0.031-1.00	351,585 (33.2)		3337 (82.1)	179750 (33.2)		1669 (81.7)
MsIsaac frailty score						
0-3	341,885 (32.2)	<.0001	318 (7.8)	188800 (34.9)	<.0001	202 (9.9)
3.5-5.5	373,570 (35.2)		625 (15.4)	183124 (33.8)		340 (16.6)
6-23	344,797 (32.5)		3122 (76.8)	169778 (31.3)		1503 (73.5)
ADG Frailty Flag	5513 (0.5)	<.0001	335 (8.2)	2642 (0.5)	<.0001	182 (8.9)
Distance‡, octiles (km)						
shortest (0-1.60)	132,271 (12.5)	<.0001	741 (18.2)	67572 (12.5)	<.0001	396 (19.4)
2nd (1.61-2.60)	132,557 (12.5)		506 (12.4)	67670 (12.5)		304 (14.9)
3rd (2.61-3.49)	132,568 (12.5)		434 (10.7)	67782 (12.5)		199 (9.7)
4th (3.50-4.54)	132,650 (12.5)		428 (10.5)	67746 (12.5)		201 (9.8)
5th (4.55-13.1)	132,602 (12.5)		438 (10.8)	67680 (12.5)		195 (9.5)

6th (13.2-47.4)	132,037 (12.5)		420 (10.3)	67823 (12.5)		225 (11.0)
7th (47.4-97.0)	133,114 (12.5)		506 (12.4)	67662 (12.5)		248 (12.1)
highest (97.1-1000)	132,453 (12.5)		592 (14.6)	67768 (12.5)		276 (13.5)
Statistical Area Classification§						
CMA	661405 (62.4)		2474 (60.9)	338647 (62.5)		1259 (61.6)
CA	100224 (9.5)		351 (8.6)	51169 (9.4)		180 (8.8)
strong MIZ	60696 (5.7)	<.0001	200 (4.9)	31806 (5.9)	0.03	97 (4.7)
mod MIZ	122798 (11.6)		507 (12.5)	62136 (11.5)		276 (13.5)
weak MIZ	98286 (9.3)		440 (10.8)	49455 (9.1)		199 (9.7)
no MIZ	16804 (1.6)		92 (2.3)	8478 (1.6)		33 (1.6)
Dementia	15968 (1.5)	<.0001	762 (18.8)	8023 (1.5)	<.0001	348 (17.0)
Elixhauser comorbidities						
Hypertension without complications	347,428 (32.8)	<.0001	2419 (59.5)	189505 (35.0)	<.0001	1311 (64.1)
Depression	232,502 (21.9)	<.0001	1132 (27.9)	120653 (22.3)	<.0001	586 (28.7)
Rheumatoid arthritis/CVD	187,681 (17.7)	<.0001	840 (20.7)	98191 (18.1)	<.0001	454 (22.2)
Diabetes mellitus without complications	146,011 (13.8)	<.0001	1286 (31.6)	78753 (14.5)	<.0001	721 (35.3)
Chronic pulmonary disorders	126,858 (12.0)	<.0001	1107 (27.2)	68258 (12.6)	<.0001	550 (26.9)
Hypothyoridism	74,779 (7.1)	<.0001	420 (10.3)	40957 (7.6)	<.0001	215 (10.5)
Cardiac arrythmia	41,327 (3.9)	<.0001	809 (19.9)	21582 (4.0)	<.0001	408 (20.0)
Deficiency anemia	31,376 (3.0)	<.0001	351 (8.6)	19401 (3.6)	<.0001	204 (10.0)
Obesity	29,413 (2.8)	<.0001	189 (4.7)	15654 (2.9)	<.0001	105 (5.1)
Other neurologic disorders	27,274 (2.6)	<.0001	453 (11.1)	14521 (2.7)	<.0001	226 (11.1)
Congestive heart failure	24,988 (2.4)	<.0001	917 (22.6)	12649 (2.3)	<.0001	414 (20.3)
Drug abuse	23,822 (2.3)	<.0001	135 (3.3)	11165 (2.1)	<.0001	70 (3.4)
Cancer without metastices	22,118 (2.1)	<.0001	368 (9.1)	11567 (2.1)	<.0001	188 (9.2)
Peripheral vascular disease	21,862 (2.1)	<.0001	433 (10.7)	11383 (2.1)	<.0001	191 (9.3)
Liver disease	19,310 (1.8)	<.0001	194 (4.8)	10618 (2.0)	<.0001	94 (4.6)
Fluid/electolyte disorders	13,979 (1.3)	<.0001	430 (10.6)	7316 (1.4)	<.0001	205 (10.0)
Psychosis	13,474 (1.3)	<.0001	352 (8.7)	7569 (1.4)	<.0001	163 (8.0)
Coagulopathy	12,663 (1.2)	<.0001	193 (4.8)	6290 (1.2)	<.0001	91 (4.5)
Renal disease	12,558 (1.2)	<.0001	422 (10.4)	6600 (1.2)	<.0001	204 (10.0)
Diabetes mellitus with complications	12,353 (1.2)	<.0001	485 (11.9)	6193 (1.1)	<.0001	238 (11.6)
Valvular heart disease	10,449 (1.0)	<.0001	197 (4.9)	5301 (1.0)	<.0001	100 (4.9)
Peptic ulcer disease without bleeding	7570 (0.7)	0.014	63 (1.6)	3744 (0.7)	<.0001	47 (2.3)
Alcohol abuse	7454 (0.7)	<.0001	138 (3.4)	3521 (0.6)	<.0001	67 (3.3)

Paraplegia/hemiplegia	4097 (0.4)	<.0001	81 (2.0)	1955 (0.4)	<.0001	42 (2.1)
Pulmonary circulatory disorders	4039 (0.4)	<.0001	99 (2.4)	2047 (0.4)	<.0001	45 (2.2)
Lymphoma	2145 (0.2)	<.0001	57 (1.4)	1156 (0.2)	<.0001	30 (1.5)
Hypertension with complications	1759 (0.2)	<.0001	44 (1.1)	1159 (0.2)	<.0001	21 (1.0)
Metastatic cancer	1235 (0.1)	<.0001	105 (2.6)	721 (0.1)	<.0001	47 (2.3)
Weight loss	1149 (0.1)	<.0001	52 (1.3)	596 (0.1)	<.0001	14 (0.7)
HIV/AIDS	809 (0.08)	0.006	8 (0.2)	642 (0.1)	0.02	6 (0.3)
Blood loss anemia	283 (0.03)	<.0001	15 (0.4)	121 (0.0)	<.0001	7 (0.3)
Hospital-days¶						
A, mean±SD	0.41 ± 3.67	<.0001	4.08 ± 12.80	0.35 ± 3.25	<.0001	3.48 ± 10.85
median (IQR)	0 (0, 0)	<.0001	0 (0, 0)	0 (0, 0)	<.0001	0 (0, 0)
B, mean±SD	0.22 ± 2.44	<.0001	2.22 ± 8.69	0.20 ± 2.32	<.0001	2.20 ± 9.06
median (IQR)	0 (0, 0)	<.0001	0 (0, 0)	0 (0, 0)	<.0001	0 (0, 0)
C, mean±SD	0.11 ± 1.61	<.0001	1.37 ± 6.41	0.11 ± 1.58	<.0001	1.52 ± 6.53
median (IQR)	0 (0, 0)	<.0001	0 (0, 0)	0 (0, 0)	<.0001	0 (0, 0)
D, mean±SD	0.097 ± 1.38	<.0001	1.47 ± 5.87	0.10 ± 1.41	<.0001	1.77 ± 6.58
median (IQR)	0 (0, 0)	<.0001	0 (0, 0)	0 (0, 0)	<.0001	0 (0, 0)
ALC+Rehabilitation-days¶						
A, mean±SD	0.17 ± 3.41	<.0001	2.55 ± 14.03	0.12 ± 2.71	<.0001	1.73 ± 12.22
median (IQR)	0 (0, 0)	<.0001	0 (0, 0)	0 (0, 0)	<.0001	0 (0, 0)
B, mean±SD	0.081 ± 2.17	<.0001	1.17 ± 8.84	0.059 ± 1.75	<.0001	0.86 ± 6.86
median (IQR)	0 (0, 0)	<.0001	0 (0, 0)	0 (0, 0)	<.0001	0 (0, 0)
C, mean±SD	0.047 ± 1.49	<.0001	0.70 ± 5.54	0.043 ± 1.42	<.0001	0.71 ± 5.77
median (IQR)	0 (0, 0)	<.0001	0 (0, 0)	0 (0, 0)	<.0001	0 (0, 0)
D, mean±SD	0.041 ± 1.34	<.0001	0.63 ± 4.91	0.038 ± 1.28	<.0001	0.76 ± 5.65
median (IQR)	0 (0, 0)	<.0001	0 (0, 0)	0 (0, 0)	<.0001	0 (0, 0)
Health Links-days¶						
A, mean±SD	0.087 ± 0.968	<.0001	0.149 ± 1.196	0.090 ± 0.907	0.002	0.153 ± 1.068
median (IQR)	0 (0, 0)	<.0001	0 (0, 0)	0 (0, 0)	0.002	0 (0, 0)
B, mean±SD	0.044 ± 0.512	<.0001	0.096 ± 0.847	0.039 ± 0.441	0.09	0.056 ± 0.462
median (IQR)	0 (0, 0)	<.0001	0 (0, 0)	0 (0, 0)	0.09	0 (0, 0)
C, mean±SD	0.022 ± 0.283	<.0001	0.041 ± 0.348	0.019 ± 0.248	0.001	0.037 ± 0.291
median (IQR)	0 (0, 0)	<.0001	0 (0, 0)	0 (0, 0)	0.001	0 (0, 0)
D, mean±SD	0.021 ± 0.252	<.0001	0.042 ± 0.365	0.020 ± 0.237	<.0001	0.043 ± 0.309
median (IQR)	0 (0, 0)	<.0001	0 (0, 0)	0 (0, 0)	<.0001	0 (0, 0)

Outpatient clinic visits¶						
A, mean±SD	5.2 ± 5.5	<.0001	7.7 ± 7.3	5.4 ± 5.5	<.0001	8.2 ± 7.3
median (IQR)	4 (1, 7)	<.0001	6 (2, 11)	4 (1, 8)	<.0001	7 (3, 12)
B, mean±SD	2.8 ± 3.1	<.0001	4.3 ± 4.4	2.8 ± 3.1	<.0001	4.4 ± 4.2
median (IQR)	2 (0, 4)	<.0001	3 (1, 6)	2 (0, 4)	<.0001	3 (1, 6)
C, mean±SD	1.3 ± 1.7	<.0001	2.1 ± 2.4	1.4 ± 1.7	<.0001	2.1 ± 2.4
median (IQR)	1 (0, 2)	<.0001	1 (0, 3)	1 (0, 2)	<.0001	1 (0, 3)
D, mean±SD	1.3 ± 1.7	<.0001	2.2 ± 2.4	1.3 ± 1.7	<.0001	2.1 ± 2.5
median (IQR)	1 (0, 2)	<.0001	1 (0, 3)	1 (0, 2)	<.0001	1 (0, 3)
Outpatient laboratory test counts¶						
A, mean±SD	4.7 ± 7.5	<.0001	7.1 ± 11.7	5.0 ± 7.9	<.0001	7.6 ± 11.7
median (IQR)	0 (0, 8)	<.0001	1 (0 11)	0 (0, 8)	<.0001	2 (0, 12)
B, mean±SD	2.5 ± 4.8	<.0001	4.0 ± 7.2	2.7 ± 5.1	<.0001	4.2 ± 7.4
median (IQR)	0 (0, 3)	<.0001	0 (0, 6)	0 (0, 4)	<.0001	0 (0, 6)
C, mean±SD	1.2 ± 3.1	<.0001	1.9 ± 4.2	1.3 ± 3.3	<.0001	1.9 ± 4.1
median (IQR)	0 (0, 0)	<.0001	0 (0, 2)	0 (0, 0)	<.0001	0 (0, 2)
D, mean±SD	1.2 ± 3.1	<.0001	2.1 ± 4.3	1.4 ± 3.3	<.0001	2.1 ± 4.5
median (IQR)	0 (0, 0)	<.0001	0 (0, 2)	0 (0, 0)	<.0001	0 (0, 2)
ATC4 prescription counts¶						
A, mean±SD	3.6 ± 3.8	<.0001	7.6 ± 5.4	3.6 ± 3.8	<.0001	7.5 ± 5.3
median (IQR)	3 (1, 5)	<.0001	7 (4, 11)	3 (1, 5)	<.0001	7 (3, 11)
B, mean±SD	2.8 ± 3.1	<.0001	6.3 ± 4.6	2.8 ± 3.2	<.0001	6.3 ± 4.5
median (IQR)	2 (0, 4)	<.0001	6 (3, 9)	2 (0, 4)	<.0001	6 (3, 9)
C, mean±SD	2.3 ± 2.8	<.0001	5.5 ± 4.2	2.3 ± 2.8	<.0001	5.5 ± 4.1
median (IQR)	1 (0, 3)	<.0001	5 (2, 8)	1 (0, 4)	<.0001	5 (2, 8)
D, mean±SD	2.3 ± 2.8	<.0001	5.6 ± 4.3	2.3 ± 2.9	<.0001	5.7 ± 4.3
median (IQR)	1 (0, 3)	<.0001	5, (2, 8)	1 (0, 4)	<.0001	5 (2, 9)

†Fiscal years (April 1-May 30); s, values omitted as representing <5 individuals or required to censor other group(s) with <5 individuals; ICU, intensive care unit; IMV, invasive mechanical ventilation; GI, gastrointestinal; ADG, Johns Hopkins Aggregated Diagnosis Group system™; ALC, alternative level of care; ATC4, fourth level of the Anatomic Therapeutic Chemical Classification system; CVD, collagen-vascular diseases

‡Distance between residence and nearest high-intensity intensive care unit

§CMA (census metropolitan area), CA (census area), MIZ (metropolitan influenzed zone)

¶Timing backwards from the Start date: (A) 13-24 months prior, (B) 5-12 months prior, (C) 4-6 months prior, (D) 0-3 months prior

Supplementary Table 5. Optimal CART tree branching, among 2644 total terminal leaves.

#branching level	# of terminal leaves at this level	% of all terminal leaves
1	1	0.04
4	8	0.30
5	17	0.64
6	33	1.25
7	61	2.31
8	71	2.69
9	78	2.95
10	81	3.06
11	133	5.03
12	104	3.93
13	138	5.22
14	192	7.26
15	150	5.67
16	133	5.03
17	123	4.65
18	124	4.69
19	136	5.14
20	146	5.52
21	113	4.27
22	126	4.77
23	86	3.25
24	123	4.65
25	83	3.14
26	106	4.01
27	88	3.33
28	63	2.38
29	63	2.38
30	64	2.42
Total	2644	100

Supplementary Table 6. Relative predictive value of all input variables in the optimal CART tree solution.

	Input Variable	*Relative Importance*
1	Socioeconomic status (SEFI-2)	1.000 (reference)
2	Lives in long-term care	0.883
3	Segal frailty score	0.879
4	Distance from home to closer of Winnipeg and Brandon	0.860
5	MsIsaac frailty score	0.810
6	Age	0.756
7	Outpatient clinic visits: 7-12 months prior to Start date	0.587
8	Outpatient clinic visits: 13-24 months prior to Start date	0.580
9	Outpatient laboratory test counts: 13-24 months prior to Start date	0.577
10	ATC4 prescription counts: 7-12 months prior to Start date	0.558
11	ATC4 prescription counts: 4-6 months prior to Start date	0.549
12	ATC4 prescription counts: 13-24 months prior to Start date	0.535
13	ATC4 prescription counts: 0-3 months prior to Start date	0.504
14	Hospital-days: 13-24 months prior to Start date	0.458
15	Outpatient laboratory test counts: 7-12 months prior to Start date	0.449
16	Outpatient clinic visits: 7-12 months prior to Start date	0.444
17	Outpatient clinic visits: 0-3 months prior to Start date	0.421
18	Outpatient laboratory test counts: 4-6 months prior to Start date	0.416
19	Hospital-days: 0-3 months prior to Start date	0.401
20	Hospital-days: 7-12 months prior to Start date	0.383
21	Hospital-days: 4-6 months prior to Start date	0.301
22	Metastatic cancer	0.285
23	Outpatient laboratory test counts: 4-6 months prior to Start date	0.274
24	Statistical area classification	0.200
25	Open homecare file	0.186
26	Diabetes mellitus with complications	0.181
27	Health Link-days: 7-12 months prior to Start date	0.178
28	Chronic pulmonary disorders	0.176
29	Alternative level of care+Rehabilitation-days: 13-24 months prior to Start date	0.160
30	Sex	0.159
31	Alternative level of care+Rehabilitation-days: 7-12 months prior to Start date	0.157
32	Timing of most recent ICU admission	0.150
33	Dementia	0.145
34	Cancer without metastases	0.144
35	Obesity	0.140
36	Congestive heart failure	0.135
37	Timing of most recent cardiac catheterization	0.134
38	Cardiac arrythmia	0.131

39	Timing of most recent GI endoscopy	0.126
40	Rheumatoid arthritis/collagen vascular disease	0.113
41	Renal disease	0.111
42	Prior income assistance	0.109
43	Hypertension with complications	0.102
44	Other neurologic disorders	0.099
45	Valvular heart disease	0.097
46	Paraplegia/hemiplegia	0.096
47	Hypertension without complications	0.094
48	Drug abuse	0.094
49	Psychosis	0.093
50	Health Link-days: 4-6 months prior to Start date	0.075
51	Weight loss	0.074
52	HIV/AIDS	0.074
53	Lymphoma	0.055
54	Alcohol abuse	0.054
55	Pulmonary circulation disorders	0.051
56	Paneled for long-term care	0.046
57	Peripheral vascular disease	0.046
58	Health Link-days: 13-24 months prior to Start date	0.045
59	Depression	0.044
60	Alternative level of care+Rehabilitation-days: 0-3 months prior to Start date	0.043
61	Coagulopathy	0.040
62	Health Link-days: 0-3 months prior to Start date	0.037
63	Fluid/electrolyte disorders	0.035
64	Liver disease	0.032
65	Deficiency anemia	0.027
66	Peptic ulcer disease without bleeding	0.026
67	Alternative level of care+Rehabilitation-days: 4-6 months prior to Start date	0
68	Blood loss anemia	0
69	Hypothyroidism	0
70	Timing of most recent bronchoscopy	0
71	Diabetes mellitus without complications	0
72	ACG frailty indicator†	0

ATC4, fourth level of the Anatomic Therapeutic Chemical Classification system; SEFI, socioeconomic factor index; †From the Johns Hopkins Adjusted Clinical Group® (ACG®) Case-Mix System

SUPPLEMENT REFERENCES

1. Lemon SC, Roy J, Clark MA, Friedmann PD, Rakowski W. Classification and Regression Tree Analysis in Public Health: Methodological Review and Comparison With Logistic Regression. *Annals of Behavioral Medicine.* 2003;23(3):172-181.
2. Wielenga D. Identifying and Overcoming Common Data Mining Mistakes SAS Institute. SAS Global Forum 2007 Web site. https://support.sas.com/resources/papers/proceedings/proceedings/forum2007/073-2007.pdf. Published 2007. Accessed May 17, 2019.
3. Sharma KS. *Predictive Modeling with SAS Enterprise Miner.* 3rd ed. Cary, NC: SAS Institute; 2017.
4. Chateau D, Metge C, Prior H, Soodeen R. Learning from the census: the Socio-economic Factor Index (SEFI) and health outcomes in Manitoba. *Canadian Journal of Public Health.* 2012;103(8 Suppl 2):S23-S27.
5. Statistical Area Classification (SAC). Statistics Canada. http://www.statcan.gc.ca/pub/92-195-x/2011001/other-autre/sac-css/sac-css-eng.htm. Published 2016. Accessed December 19, 2016.
6. Elixhauser A, Steiner C, Harris DR, Coffey RM. Comorbidity Measures for Use with Administrative Data. *Medical Care.* 1998;36(1):8-27.
7. Quan H, Sundararajan V, Halfon P, et al. Coding Algorithms for Defining Comorbidities in ICD-9-CM and ICD-10 Administrative Data. *Medical Care.* 2005;43(11):1130-1139.
8. Segal JB, Chang H-Y, Du Y, Walston JD, Carlson MC, Varadhan R. Development of a Claims-based Frailty Indicator Anchored to a Well-established Frailty Phenotype. *Medical Care.* 2017;55(7):716-722.
9. McIsaac DI, Wong CA, Huang A, Moloo H, van Walraven C. Derivation and Validation of a Generalizable Preoperative Frailty Index Using Population-based Health Administrative Data. *Annals of Surgery.* 2019;270(1):102-108.
10. *The Johns Hopkins ACG® System: Technical Reference Guide Version 9.0.* Baltimore: Johns Hopkins University; 2009.
11. Anatomical Therapeutic Chemical (ATC) Classification. World Health Organizations. https://www.who.int/medicines/regulation/medicines-safety/toolkit_atc/en/. Published 2021. Accessed June 28, 2020.
12. Manitoba Health Links. Manitoba Health. https://centredesante.mb.ca/resources/in-your-community/health-links-info-sante/?lang=en. Published 2020. Accessed April 4, 2020.
